# Impaired Glucose Metabolism in Bipolar Patients: The Role of Psychiatrists in Its Detection and Management

**DOI:** 10.3390/ijerph16071132

**Published:** 2019-03-29

**Authors:** Dorota Łojko, Maciej Owecki, Aleksandra Suwalska

**Affiliations:** 1 Department of Adult Psychiatry, Poznan University of Medical Sciences, 61-701 Poznan, Poland; 2 Department of Public Health, Chair of Social Medicine, Poznan University of Medical Sciences, 61-701 Poznan, Poland; mowecki@ump.edu.pl; 3 Department of Mental Health, Chair of Psychiatry, Poznan University of Medical Sciences, 61-701 Poznan, Poland; asuwalska@ump.edu.pl

**Keywords:** bipolar disorder, insulin resistance, type 2 diabetes, TyG, HOMA, impaired glucose metabolism, comorbidity

## Abstract

Bipolar patients have a higher risk of type 2 diabetes and obesity, which are associated with cardiovascular diseases as the leading cause of death in this group. Additionally, there is growing evidence that impaired glucose metabolism in bipolar patients is associated with rapid cycling, poor response to mood stabilizers and chronic course of illness. The aim of the study was to assess the prevalence of type 2 diabetes and other types of impaired glucose metabolism in bipolar patients along with an evaluation of the Fasting Triglycerides and Glucose Index (TyG) as a method of the insulin sensitivity assessment. The analysis of fasting glycemia, insulinemia and lipid profile in euthymic bipolar patients was performed, and the Homeostasis model assessment for insulin resistance (HOMA-IR) and TyG were computed. Type 2 diabetes was observed in 9% and insulin resistance with HOMA-IR in 48% of patients. The TyG and HOMA-IR indices were correlated (*p* < 0.0001), the TyG index value of 4.7 had the highest sensitivity and specificity for insulin resistance detection. The usefulness of TyG in the recognition of insulin resistance in bipolar patients was suggested. The significant role of psychiatrists in the detection and management of impaired glucose metabolism in bipolar patients was presented.

## 1. Introduction

According to the literature, the risk of type 2 diabetes is up to three times higher in patients with bipolar disorder than in healthy controls of similar age and sex [1,2,3]. Over half of bipolar disorder (BD) patients have impaired glucose metabolism, including insulin resistance (IR), impaired glucose tolerance or type 2 diabetes (T2D) [4,5]. This is not entirely related to side effects of pharmacological treatment as even in drug-naïve BD patients IR has been significantly more prevalent than in a healthy population [6]. It is well known that metabolic disturbances usually progress from euglycemia to dysglycemia, insulin resistance and glucose intolerance and in some patients eventually (but not inevitably) to T2D with all its serious consequences and increased mortality rates [7]. Insulin resistance and hyperinsulinemia are also related to the high death rate, due to coronary vascular disease in nondiabetic subjects [8].

Additionally, there is growing evidence that comorbidity of T2D or insulin resistance in bipolar patients is frequently associated with a poor response to mood stabilizing treatment, increased prevalence of rapid cycling and a chronic course of BD. When compared with euglycemic patients, BD patients with T2D or insulin resistance had three times higher odds of a chronic course of bipolar disorder, three times higher odds of rapid cycling and were more likely to be refractory to lithium treatment [4]. Mansur et al. have shown that T2D or impaired fasting glucose negatively moderate the course of illness in bipolar patients [5] and Cairns et al. in their recently published paper [9] have pointed out to the worsening of BD course after the onset of insulin resistance. Moreover, impaired glucose metabolism may influence brain structure and function as peripheral insulin resistance is associated with reduced global and regional brain glucose metabolism [10] and with the restrain of insulin transport into the brain [11]. Compared to euglycemic BD subjects, individuals with comorbid BD and T2D have been shown to have smaller hippocampal and cortical volumes, as well as neurochemical abnormalities, indicative of neuronal/axonal loss. Both T2D and impaired fasting glucose may be risk factors for hippocampal and cortical brain alterations in BD [12]. Such structural and metabolic brain changes in BD patients with IR/T2D are associated with cognitive impairment [12,13,14].

Whereas many aspects of impaired glucose metabolism (including pathophysiology of insulin sensitivity) and its relationships to BD morbidity, course and treatment remain to be elucidated, long-term complications (in both somatic and psychiatric health) of IR/2TD point to the necessity of detection of impaired glucose metabolism at the stage of insulin resistance, which may appear 10–20 years before the final diagnosis of T2D [15,16,17].

The diagnosis of type 2 diabetes proposed by the World Health Organization [18] and the American Diabetes Association [19] is based on fasting glucose level in excess of 125 mg/dL (7 mmol/L), but still there is neither a consensus nor recommendations with regard to the methods and cut-off points for the detection of IR as an early phase of impaired glucose metabolism. Insulin resistance, in other words, reduced insulin sensitivity is a pathological state characterized by a lack of physiological response of peripheral tissues to insulin action, which may lead to various disturbances (i.e., dyslipidemia, hypertension, T2D, abdominal obesity, hypercoagulability and defects in the fibrinolytic system, fatty liver, and an increased risk of coronary heart disease) [20].

The gold standard for the determination of insulin sensitivity is the euglycemic-hyperinsulinemic clamp which directly measures the glucose uptake in peripheral tissues in vivo in the situation of elevated insulin concentrations [21]. As it is a time-consuming, complex, invasive and expensive method, more clinically accessible techniques for the estimation of insulin sensitivity are needed. Several methods of insulin sensitivity assessment based on the computed indices (mathematical formulas) derived from fasting insulin, glucose, triglycerides, post-loaded glucose/insulin levels have been proposed [22]. A test that would identify an insulin-resistant individual has to be practical, inexpensive and easy to perform. The Homeostasis Model Assessment (HOMA) is a well-known and widely used validated method to measure insulin sensitivity based on fasting serum insulin and glucose levels [23]. HOMA insulin resistance index (HOMA-IR) highly correlates with the insulin sensitivity assessed by the euglycemic–hyperinsulinemic clamp in subjects with various degrees of glucose tolerance [24]. However, there is still no universal cut-off for HOMA-IR which depends on the studied population [25]. In 2008, the Fasting Triglycerides and Glucose (TyG) index was proposed and validated by Simental-Mendia et al. [26,27] as a new formula to evaluate IR from the levels of serum triglycerides and fasting glucose. TyG index results correlate with results of the euglycemic-hyperinsulinemic clamp technique in the assessment of insulin sensitivity [27] and have been found to be useful in the identification of subjects at risk of IR/T2D in adolescent and adult populations [22,28,29].

When using surrogate fasting indices of IR one should be aware of their limitations. Computed indices are always a simplification of very complex glucose homoeostasis assessment and small, but clinically relevant, variations in insulin sensitivity may be overlooked by simple indices. The clamp test makes use of the serial sampling of arterialized blood, for computed indices clinicians use one vein blood sample for insulin level assessment [23]. Such a single insulin sample is used in practice despite the original recommendation [23] to use the mean of three samples taken at 5 min intervals (due to the fact that insulin secretion follows a pulsatito tale pattern). The usefulness of HOMA-IR is limited in pathophysiological situations, such as insulinoma and primary hyperaldosteronism [22]. HOMA-IR fails to provide insight into the stimulated glucose and insulin systems: It only informs what happens with the homeostatic mechanisms in the fasting state, largely reflecting insulin’s effect on hepatic glucose production and not on peripheral glucose uptake, which is the more relevant aspect concerning insulin action/resistance [30]. The TyG index does not reflect the physiological condition of constant changes in glucose and insulin either. While measuring TYG, the variability of the triglyceride and insulin levels associated with ethnicity requires further research [27].

Although validation studies of surrogate indices in various populations are limited, insulin sensitivity/resistance assessed by these indices have been shown to be relevant in the prediction of type 2 diabetes [31] or occurrence of cardiovascular diseases (CVD) [32]. Bearing in mind the fact that computed IR does not unambiguously classify a patient as insulin-resistant or insulin-sensitive (no reference values have been established), the clinician can use this measure in combination with the assessment of the classical risk factors associated with various definitions of the metabolic syndrome (such as blood pressure, lipid profile, BMI and waist circumference) in the monitoring of glucose metabolism impairments [22].

Patients with BD are affectively symptomatic for almost half of their lives [33]. Psychiatrists and other mental health professionals define their treatment objectives as a reduction of the severity of affective symptoms, achieving full sustained remission, and good social, professional, family functioning. Bipolar disorder is mainly managed using pharmacological treatment combined with psychoeducation and, in many cases, with a cognitive-behavioral therapy aimed at coping with illness. Clinical treatment guidelines also point to the importance of assessment of the physical health of bipolar patients as they have an increased risk of premature mortality [34], mainly due to cardiovascular diseases, in a large part related to weight gain. Treatment recommendations in bipolar disorder refer to weight gain control, and management mainly in terms of side effects of psychotropic medication. Similarly, common medical comorbidities, such as metabolic syndrome and T2D are considered to be associated with medications used in BD.

In case of rapid or significant weight gain, it is recommended to take into account the effects of medication, and assess the role of the mental state, somatic and lifestyle factors in their development [35]. Regular monitoring of weight, abdominal circumference, blood pressure, serum lipids and glucose on—(at least) annual basis is recommended in practice guidelines [35,36]. In the recently published Canadian guidelines for the management of patients with bipolar disorder [37] the role of glucose/carbohydrate metabolism was mentioned, as for the first time the contribution of insulin dysfunction to BD pathophysiology was noted.

Despite a growing body of evidence confirming the twofold influence of impaired glucose metabolism in bipolar disorder, it remains to be under-detected and undertreated. There are no guidelines concerning the coexistence of insulin resistance and BD and no recommendations to screen affective patients for insulin resistance at an early stage of impaired glucose metabolism.

The aim of this study was to assess the prevalence of type 2 diabetes and other types of impaired glucose metabolism in bipolar patients along with the evaluation of TyG index as a method of insulin sensitivity assessment. The procedures and the twofold reason for early detection and monitoring of impaired glucose metabolism in bipolar patients were presented.

## 2. Materials and Methods

Patients with bipolar disorder treated for more than five years in an outpatient university clinic were screened for the study. Prior to enrolment patients were diagnosed by two psychiatrists according to the International Statistical Classification of Diseases and Related Health Problems, 10th Revision—(ICD-10) criteria [38]. At the time of the assessment, all patients were euthymic for at least four months, as defined by a score of ≤7 on the 17-item Hamilton Depression Rating Scale [39], and a score of ≤7 on the Young Mania Rating Scale [40]. Patients with a history of head injury, epilepsy, diagnosis of dementia, mental retardation or any severe/unstable somatic diseases, bariatric treatment or taking lipid-lowering medications, steroids or immunosuppression were excluded from the study. All enrolled patients were taking mood stabilizers, i.e., lithium and/or valproic acid, as well as atypical antipsychotics; all of them had been treated with antidepressants in the past. 

The study was conducted in accordance with the Helsinki Declaration and the protocol was approved by the Ethics Committee of Poznan University of Medical Sciences (Resolution no. 121/13). All subjects gave their written consent after receiving a full explanation of the nature and procedures of the study.

Blood samples were collected between 8:00 and 9:00 a.m. after an overnight fast of at least 9 h. Fasting serum triglycerides and glucose were assayed using colorimetric enzymatic methods in an automated clinical chemistry system (Olympus AU 680 Chemistry Analyzer Beckman Coulter, Brea, CA, USA). Insulin levels were measured by chemiluminescence immunoassay (ARCITECTi 1000SN immunoassay analyzer, Abbott Diagnostics, Chicago, IL, USA). Samples were analyzed in the same laboratory with the same assay to decrease variability.

Insulin sensitivity was estimated using two mathematically computed models: Homeostasis model assessment for insulin resistance (HOMA-IR) and Fasting Triglycerides and Glucose (TyG) Index. Homeostasis model assessment [23] is a validated method used to quantify insulin resistance from fasting glucose and insulin concentrations. HOMA-IR is calculated using the following formula: HOMA-IR = (fasting insulin × fasting glucose)/22.5 [20]. HOMA-IR values of 2.0 or more have been previously suggested as indicating insulin resistance, based on the point at which the risk of metabolic syndrome significantly increases [41,42,43] or the highest quartile of the HOMA-IR index, as measured in non-diabetic subjects [25,44]. We used a HOMA-IR value of 2.0 and more to establish insulin resistance, like in the previous study on BD patients [4].

Based on the fasting glucose and triglycerides levels, TyG Index was computed as the natural logarithm (Ln) of ((fasting triglycerides) (mg/dL) multiplied by fasting glucose (mg/dL)/2) [26,45].

In all studied subjects, weight was measured in light clothing without shoes to the nearest 0.1 kg (electronic scale WPT 100/200 RadWag Poland). Bodyweight status was described by the body mass index (BMI), expressed as the weight in kilograms divided by the square of the height in meters (kg/m^2^). Waist circumference was measured with a retractable measuring tape for precise body measurements over the unclothed abdomen at the narrowest point between the lower rib and iliac crest [46]. A semi-structured questionnaire developed by authors of the study (A.S. and D.Ł.) was used, including socio-demographic information data, information on educational status (number of years of education), living alone or with family, concomitant diseases and lifestyle.

Statistical analysis. To evaluate the normality of the distribution of the variables, the Kolmogorov-Smirnov test was applied. The sociodemographic, lifestyle, and clinical characteristics of the subjects were compared using the Mann-Whitney test for continuous variables and the *χ^2^* test for categorical variables. Spearman’s rank correlation coefficient was used for interdependence analysis for continuous variables. Sensitivity and specificity of the TyG index were estimated as a function of the threshold used to define insulin resistance by the HOMA. The optimal value of the TyG index for insulin sensitivity cut off point was established on a receiver operating characteristic (ROC) scatter plot. The area under the ROC curve (AUC), as a summary of the overall diagnostic accuracy of the test, was estimated. The level of statistical significance was set at *p* < 0.05. SPSS (IBM Corp. Released 2016. IBM SPSS Statistics for Windows, Version 24.0. IBM Corp, Armonk, NY, USA) was used for all analyses. Continuous variables are presented as mean values and standard deviation, while categorical variables are presented as a sample percentage (%).

## 3. Results

The group of 104 bipolar patients was screened. Nine patients did not meet the inclusion criteria, four participants were excluded from analysis, due to missing data and three patients withdrew their consent. Five of the 88 patients had been diagnosed with T2D prior to the study, and in three of the 88 subjects, diabetes was detected based on the criterion of random fasting glucose level obtained by venous blood draw in excess of 125 mg/dL (7 mmol/dL) [47] confirmed by repeated abnormal fasting glucose level on another day. The general characteristics of the final studied group and laboratory tests results are shown in Table 1.

**Figure 1:**
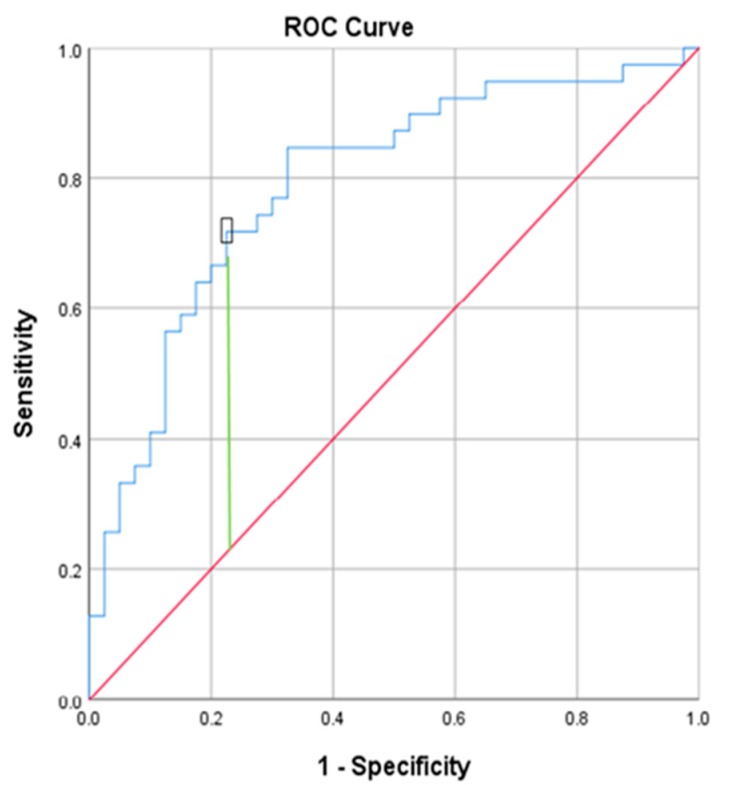
ROC curve sensitivity represents true-positive results and 1—specificity, the false positive results. The best TyG index value indicating insulin resistance was 4.695, Youden Index = 0.49.

Impaired fasting glycemia (IFG) defined as 100–125 mg/dL (5.6–6.9 mmol/dL) [47] was identified in 20 patients. To assess insulin resistance, HOMA-IR and TyG indices were computed for each patient according to the formulas described above. HOMA-IR equal to or above 2 (which indicated insulin resistance) was noted in 42 patients, and 64% of them (27 subjects) had fasting glucose level below 100 mg/dL (5.6 mmol/L). To find cut–off points for TyG index patients were divided into two groups based on the HOMA-IR value (less than 2 and 2 and above). The values of TyG were compared between the groups and statistically significant differences between them were found (Mann-Whitney’s test U, *p* < 0.0001). A ROC analysis was carried out for TyG. The ROC curve shows the relationship between sensitivity and specificity. On the x-axis, there are 1-specificity values and on the y-axis, there are sensitivity values. The obtained points were connected to each other. The resulting curve, and more precisely the field under it, illustrates the classification quality of the TyG variable. The area under the curve (AUC) is 0.787 and is significantly different from 0.5 (*p* < 0.0001). The 95% confidence interval is (0.686; 0.887), it does not contain 1, which proves that the TyG parameter differentiates well between both analyzed groups. Based on the Youden index, a TyG cut-off of 4.695 was determined. For this TyG value, Youden index = 0.49, the sensitivity is 0.738, and the specificity is 0.756. The TyG value of 4.7 determined in the studied bipolar group was the cuf-off point for insulin resistance (Figure 1).

## 4. Discussion

In our study, more than half of the bipolar patients had impaired glucose metabolism, i.e., insulin resistance, impaired fasting glycemia or type 2 diabetes. These results are comparable with other reports. In 10.2% of patients type 2 diabetes was observed, which was similar to 11.6% in Ruzickova’s et al. study [48], but lower compared to the results of another Canadian study [4]. The fasting glucose test revealed T2D in three patients and impaired fasting glycemia in 20 persons. Those patients were referred to as diabetologists collaborating with our department. Assessment of insulin resistance with HOMA-IR revealed it in 51% BD patients, more than the 32% reported in the previous work, in which the same conservative cut-off point has been used [4]. More than half IR patients had the fasting glucose result below 100 mg/dL, which suggests that a simple assessment of fasting glucose is not sufficient to detect impaired glucose metabolism.

In our BD group the TyG index significantly correlated with the HOMA-IR result, suggesting that it could be useful in the recognition of IR among BD subjects. The TyG index measurements, i.e., glucose and triglycerides, are available in all clinical laboratories and are part of routine laboratory assessment. Using TyG index as the insulin resistance index does not require the measurement of serum insulin levels (an expensive test that is not available in many laboratories). Thus, TyG index could be an accessible and reliable test for the detection of insulin resistance in BD. As far as we know, in our study the TyG index was used for the first time to assess the insulin resistance in bipolar patients. The best TyG index value for detection of insulin resistance in the BD group was 4.7, which showed the highest sensitivity (73.8%) and specificity (75.65%). High sensitivity indicates a low rate of false negative results, i.e., subjects with IR can be accurately identified. In a non-psychiatric population, TyG index used for the detection of insulin resistance has been 4.65 [26], 4.68 [27,49], or even less, as a value above 4.5 has been proposed by Salazar et al. [50]. We are aware that assessing insulin sensitivity using mathematical formulas is not an optimal approach as they do not provide the same degree of information as the euglycemic-hyperinsulinemic clamp, even though good concordance rates between these computed indices and the more reliable tests have been found in several studies. The best way to assess the usefulness of TyG in insulin resistance assessment in bipolar patients would be a comparison of the gold standard euglycemic-hyperinsulinemic clamp test and the TyG index in subjects with bipolar disorder. As mentioned above, clamp assessment has a number of limitations (mainly its cost and invasiveness) and can be used in studies with a small number of subjects, but is not suitable for larger groups (for clinical purposes or in epidemiological studies) and as far as we know, it has not been performed so far on bipolar patients. Therefore, in our opinion, a simple and inexpensive tool, such as TyG index in bipolar patients may contribute to the identification of insulin resistance patients with high T2D risk.

The body mass index widely used as a simple measure for defining obesity (a risk factor for T2D) may be misleading, especially in older people and in some ethnic groups. The waist circumference is well established as a good predictor of cardiovascular diseases and diabetes and considered more reliable than BMI [51,52]. Moreover, there is a subpopulation of non-overweight individuals with metabolic disturbances called metabolically abnormal normal weight or normal weight obesity. In this subpopulation measuring BMI and waist circumference is not applicable, and those individuals could be identified based on lipid profiles and/or the TyG index [53]. Such normal weight obese persons with TyG index levels above cut-off may have an approximately twofold higher risk of developing diabetes compared with metabolically healthy normal weight subjects [53] and two-thirds of them are developing obesity over ten years, with its putative consequences, including T2D and CVD [54,55]. The TyG index seems to be a useful predictor of such potential development of T2D [49,56].

Taking into account both psychiatric and somatic sequelae of impaired glucose metabolism in bipolar patients, prevention of these disturbances and their early detection and treatment are necessary. BD is a lifetime disease, and patients remain in psychiatric care, many of them on a regular basis with regularly scheduled appointments. We fully support Calkin et al. [14] who have postulated that psychiatric outpatient visits should include screening towards impaired glucose metabolism. In our opinion such screening should cover at least questions concerning the previous diagnosis of diabetes and the waist circumference measurement along with routine laboratory measurements allowing detection of type 2 diabetes, impaired fasting glucose and the TyG index calculation as a measure of IR. So far, impaired glucose metabolism assessment has often been neglected in routine psychiatric practice: The waist circumference or BMI, if assessed at all, are usually based on patients’ statements (which are not always reliable). Psychiatrists should take part in the monitoring of metabolic abnormalities, screen for impaired glucose metabolism and collaborate with other physicians when the treatment of any type of impaired glucose metabolism is required [4,57]. In turn, physicians (general practitioners, diabetologists) need to collaborate with mental health professionals to get the knowledge and experience in the management of BD patients who have a high rate of impaired glucose metabolism comorbidity. In the care of patients with comorbid glucose metabolism impairments and BD, psychiatrists should always obtain copies of results with information about the somatic status of their bipolar patients from other physicians [39]. There is a group of bipolar patients for whom a psychiatrist is the only doctor they see, thus a psychiatrist is responsible for laboratory tests and somatic state monitoring [58]. The psychiatrists should be familiar with various management issues concerning T2D [59,60,61] to help BD patients follow diabetologists’, dietitians’ and other professionals’ recommendations. In the clinical management of BD patients, pharmacological treatment should be carefully selected taking into consideration its influence on the patients’ weight and metabolic profile. In all bipolar patients, especially those requiring treatment with weight enhancing psychotropic medication, diet intervention and physical activity should be prophylactically implemented.

Clinical guidelines focus on the detection of T2D in bipolar patients, especially those who are obese and treated with atypical antipsychotics. In our opinion, much effort should be made to detect glucose metabolism impairment a step before, at the hyperglycemia and/or insulin resistance stages. As insulin resistance is a modifiable state, the prophylaxis is especially important for the outcomes of BD patients. Effects of modification of glucose metabolism on the course of bipolar illness have been reported and insulin resistance is considered as a negative factor, worsening the course of the disease and contributing to treatment resistance in BD [5,9]. Impaired glucose metabolism represents a potential risk factor for neurochemical alterations in the central nervous system in BD patients [12]. Since T2D-related neuronal tissue damage is preventable [13,62] an early diagnosis and treatment of glucose metabolism impairment are crucial. In routine BD care, the psychiatrists can and should do simple and important preventive interventions. It is essential to discuss the rules a balanced diet, encourage healthy eating habits and explain the basic principles of the diet, as the dietary habits of BD patients are often unhealthy [63]. As exercise is central to effective prevention of T2D, psychiatrists should also recommend physical activity. It is known that adults who maintain a physically active lifestyle can reduce their risk of developing impaired glucose tolerance, insulin resistance and T2D [64]. Although pharmacological therapies for prevention of T2D have not yet been recommended by the American Diabetes Association [59] and have not been approved by the U.S. Food and Drug Administration, insulin-sensitizing agents have been found to reduce T2D risk by 31–77% [65]. Notice should be taken of the first studies on the adjunctive use of pharmacological agents which influence insulin sensitivity in BD. In bipolar depression, open-label adjunctive treatment with insulin-sensitizing pioglitazone has been associated with improvement in depressive symptoms and reduced cardiometabolic risk [66].

We are aware of the limitations of this study. First of all, it is its cross-sectional character, which does not show the direction and nature of the interdependencies between the studied parameters. Our sample was derived from a tertiary referral center and it might represent a population of patients with more severe BD who might have higher rates of impaired glucose metabolism. There are also limitations of using surrogate IR indices as was noted above. We did not analyze pharmacological treatment as insulin resistance and type 2 diabetes in bipolar patients are not entirely associated with the effects of psychiatric medications [5,67,68,69] and our aim was to focus on the procedures and methods of detection of impaired glucose metabolism. Future research is required to study the relationship between impaired glucose tolerance and insulin resistance, dysregulation of the HPA axis, glucocorticoid resistance and increased proinflammatory cytokine production across both phases of bipolar disorder [70]. Despite the limitations we hope we managed to supplement data on bipolar disorder, as it was the first European study assessing insulin resistance with TyG index in euthymic BD patients.

## 5. Conclusions

More than half of BD patients in the studied group had impaired glucose metabolism i.e., type 2 diabetes, impaired fasting glucose level and/or insulin resistance. Our study assessing insulin resistance with TyG index in euthymic BD patients showed its high sensitivity and specificity for insulin resistance detection. As the TyG index is widely available and inexpensive, we hope that our findings would add some practical knowledge to everyday mental health care practice, and encourage psychiatrists to screen for glucose metabolism disturbances with this simple method.

Including screening towards impaired glucose metabolism into routine BD clinical practice, increased awareness of the detrimental effect of impaired glucose metabolism on the health state (somatic and psychiatric) and long-term consequences of undetected and untreated impaired glucose metabolism condition are of crucial importance. As the trajectory from dysglycemia to diabetes and its consequences may take many years and the process is reversible to some point, simple preventive measures and the earliest possible detection of impaired glucose metabolism may alleviate the psychiatric (poor course of illness, refractoriness to treatment, cognitive dysfunctions), as well as somatic consequences (increased risk of cardiovascular diseases and premature death).

## Figures and Tables

**Table 1:** Demographic, clinical, anthropometric characteristics and laboratory tests results of euthymic bipolar patients. The results are presented as mean and standard deviation (SD) or percentage.

	Whole Group	Men	Women	*p* *
Age (years)	58.1 (11.7)	61.8 (15.7)	56.9 (12.1)	NS
Education (years)	14.3 (7.3)	13.7 (3.3)	13.5 (3.1)	NS
Current smokers (%)	36.7	14.3	22.6	NS
Living alone (%)	32	14.3	17.7	NS
BD duration (years)	23.3 (11)	30 (18)	21 (13)	0.002 ^1^
BMI (kg/m^2^)	27.6 (5.8)	27.3 (5.9)	27.8 (5.1)	NS
25 < BMI < 30 (%)	37.6	61.9	31.1	0.01 ^2^
BMI ≥ 30 (%)	22.3	14.3	32.8	NS
Waist circumference (cm)	93.8 (14)	96.6 (13.1)	90.3 (9.3)	NS
Serum glucose level (mg/dL)	95.6 (15.4)	99.8 (22.3)	94.2 (21.1)	NS
Serum triglycerides level (mg/dL)	148.8 (81)	138.5	151.4	NS
Insulin (mU/mL)	11.9 (4)	10 (6.2)	12.8 (8.1)	NS
HOMA-IR	3.0 (0.3)	2.7 (0.3)	3.2 (0.3)	NS
TyG	4.7 (0.8)	4.7 (0.7)	4.7 (0.9)	NS

* Difference between men and women; HOMA-IR—homeostasis model assessment for insulin resistance; TyG—fasting triglycerides and glucose index, BMI—body mass index; ^1^ Mann Whitney test; ^2^ chi-square test.
